# Patienten- und Qualitätsmerkmale bei der Behandlung mit Disulfiram („Antabus“) im deutschsprachigen „Netzwerk alkoholaversive Pharmakotherapie“

**DOI:** 10.1007/s00115-024-01714-5

**Published:** 2024-08-22

**Authors:** Ulrich S. Zimmermann, Clemens Plickert, Christel Lüdecke, Markus Stuppe, Christian Rosenbeiger, Yvonne Krisam, Tobias Link, Jean Keller, Gero Bühler, Deborah Scholz-Hehn, Ursula Havemann-Reinecke, Dirk Wedekind, Mathias Luderer, Maik Spreer

**Affiliations:** 1 Medizinische Fakultät Carl Gustav Carus, Technische Universität Dresden, Dresden, Deutschland; 2 kbo Isar-Amper-Klinikum Region München, Klinik für Suchtmedizin und Psychotherapie, Ringstr. 1, 85540 Haar bei München, Deutschland; 3 Københavns Kommune, Socialforvaltning, Center for Alkohol- og Stofbehandling, Københavns, Dänemark; 4 Asklepios Fachklinikum Göttingen, Göttingen, Deutschland; 5 Helios Kliniken Schwerin, Klinik für Abhängigkeitserkrankungen, Schwerin, Deutschland; 6 Klinik für Abhängiges Verhalten und Suchtmedizin, Zentralinstitut für Seelische Gesundheit Mannheim, Mannheim, Deutschland; 7 Psychiatrisches Zentrum Nordbaden, Wiesloch, Deutschland; 8 Klinikum Ingolstadt, Zentrum für psychische Gesundheit, Ingolstadt, Deutschland; 9 Klinik für Psychiatrie, Psychotherapie und Psychosomatik, Agaplesion Markus Krankenhaus Frankfurt a. M., Frankfurt a. M, Deutschland; 10 Klinik für Psychiatrie und Psychotherapie, Universitätsmedizin Göttingen, Göttingen, Deutschland; 11 Universitätsklinikum, Klinik für Psychiatrie, Psychosomatik und Psychotherapie, Goethe-Universität Frankfurt, Frankfurt a. M., Deutschland; 12 Klinik für Psychiatrie und Psychotherapie, Universitätsklinikum Dresden, Dresden, Deutschland

**Keywords:** Alkoholabhängigkeit, Rückfallschutz, Aversivreaktion, Acetaldehyd-Dehydrogenase- Inhibitor, Anticraving, Alcohol use disorder, Protection against relapse, Aversive reaction, Acetaldehyde dehydrogenase inhibitor, Anticraving

## Abstract

**Hintergrund:**

Seit mehr als 10 Jahren ist Disulfiram in Deutschland nicht mehr zugelassen. Dennoch wird es seitens einer Reihe von Ambulanzen psychiatrischer Fachkliniken sowie niedergelassener Ärzte weiter off-label eingesetzt. Diese haben sich 2018 zum „Netzwerk alkoholaversive Pharmakotherapie“ (NAP) zusammengeschlossen, um die Qualität dieser Behandlungsform aufrechtzuhalten.

**Ziel der Arbeit:**

Beschreibung des gegenwärtigen Behandlungsumfangs, der Patientencharakteristika, der Nebenwirkungen und der begleitenden Therapieangebote der Behandlung mit Disulfiram.

**Material und Methoden:**

Seit 2019 führt das NAP in den beteiligten Zentren jährlich eine retrospektive Erhebung zu oben genannten Parametern durch.

**Ergebnisse:**

Im Zeitraum von 2019 bis 2023 wurden durch 33 Zentren zusammen 1579 Behandlungsfälle erfasst. Bei 152 Patienten wurden insgesamt 241 Trinkereignisse beschrieben, von denen 26 zu stationärer Behandlung ohne Komplikationen oder anhaltende Gesundheitsschäden führten. Häufigste Nebenwirkungen waren in absteigender Reihenfolge Körpergeruch (2,5 %), Müdigkeit, männliche sexuelle Funktionsstörungen, benigner Transaminasenanstieg, allergische Hautreaktionen und Polyneuropathie (0,8 %). Über ein Viertel der Patienten litt komorbid an Depressionen und je ca. 5 % an ADHS, emotional instabilen bzw. anderen Persönlichkeitsstörungen, Traumafolgestörungen sowie Angststörungen. 33 % der Patienten wurden mit Antidepressiva und 12 % mit sedierenden Antipsychotika behandelt. Begleitende Gruppentherapien wurden bei 66 % der Patienten angeboten.

**Diskussion:**

Die Behandlung mit Disulfiram ist legal möglich, allgemein gut verträglich und sicher. Sie wird in den meisten Behandlungszentren als Bestandteil eines Gesamtbehandlungsplanes angeboten, der eine multimodale Behandlung komorbider psychiatrischer Erkrankungen miteinschließt.

**Zusatzmaterial online:**

Zusätzliche Informationen sind in der Online-Version dieses Artikels (10.1007/s00115-024-01714-5) enthalten.

## Abstract (en)

**Background:**

More than a decade ago disulfiram lost its approval for use in Germany. Nonetheless, a considerable number of psychiatric hospital outpatient departments as well as practicing physicians continue to prescribe it. These professionals have formed the “Network for Alcohol Aversive Pharmacotherapy” (NAP) to maintain a high quality of this treatment approach.

**Objective:**

To describe the current use of disulfiram with respect to patient numbers and characteristics, side effects, and use of concomitant multimodal treatment forms.

**Material and methods:**

Since 2019 the NAP has conducted an annual retrospective survey among its members regarding the aforementioned parameters.

**Results:**

From 2019 to 2023 a total of 1579 treatment cases were described by 33 centers, 152 patients reported a total of 241 drinking events, 26 of them resulting in hospitalization but none causing complications or permanent harm. The most frequent side effects, in descending order, were unpleasant body odor (2.5%), fatigue, male sexual dysfunction, mildly elevated liver enzymes, allergic skin reactions and polyneuropathy (0.8%). More than one quarter of the patients suffered from comorbid depression, and approximately 5% from ADHD, borderline or other personality disorders, trauma-related disorders and anxiety disorders, respectively. Of the patients 33% were treated with antidepressants and 12% with sedating antipsychotics. Various forms of concomitant group therapy were offered to 66% of the patients.

**Conclusion:**

Treatment with disulfiram is legally possible, generally well-tolerated and safe. It is offered in most treatment centers as part of a comprehensive treatment plan that includes multimodal treatment of comorbid psychiatric disorders.

## Hintergrund und Fragestellung

Disulfiram inhibiert die Acetaldehyd-Dehydrogenase, was beim Konsum von Alkohol zu einer Unverträglichkeitsreaktion führt [13]. In beiden deutschen Staaten wurde dieses Wirkprinzip über viele Jahrzehnte regelmäßig eingesetzt. Beispielgebend konnte im Göttinger Projekt zur ambulanten Langzeitintensivtherapie für Alkoholkranke (ALITA) mit dem Einsatz von Disulfiram selbst bei hoch rückfallgefährdeten Patienten jahrelange Abstinenz erreicht werden [9, 10]. Weitere deutsche Originalarbeiten beschrieben gute Erfahrungen mit Disulfiram bei opioidsubstituierten Patienten [17] sowie beim Einsatz durch eine nichtakademische Versorgungsklinik [7]. Die klinische Evidenz zur Wirksamkeit wurde in mehreren systematischen Übersichtsarbeiten zusammengefasst [1, 4, 15, 16]. In einer Netzwerkmetaanalyse zum Vergleich mehrerer Medikamente zeigte sich Disulfiram bei supervidierter Vergabe zur Unterstützung der Abstinenz sowie zur Reduktion schweren Trinkens besser wirksam als Acamprosat und Naltrexon [1]. Auch in einer Metaanalyse direkter Vergleichsstudien zwischen Disulfiram und Acamprosat bzw. Naltrexon erwies sich Disulfiram jeweils als statistisch signifikant besser wirksam [16]. Die aktuelle S3-Leitlinie „Screening, Diagnose und Behandlung alkoholbezogener Störungen“ empfiehlt: „In der Postakutbehandlung außerhalb der stationären Rehabilitation kann eine pharmakotherapeutische Behandlung mit Disulfiram im Rahmen eines Gesamtbehandlungsplans angeboten werden, wenn andere zugelassene Therapieformen nicht zum Erfolg geführt haben“. (Empfehlungsgrad 0, Level of Evidence: 1b; [5]).

Angesichts dieser Fülle von Erfahrung erstaunt es, wie wenig Einigkeit zu praktischen Fragen wie Indikationen und Kontraindikationen, Dosierung, Einnahmefrequenz oder Behandlungsdauer besteht. Ein nicht einheitlich gehandhabter Aspekt ist beispielsweise die Durchführung der supervidierten Einnahme, da Disulfiram nur dann signifikant besser als die jeweilige Kontrollbehandlung wirkt, wenn die Einnahme im Rahmen eines persönlichen therapeutischen Kontaktes stattfindet [16]. Der hochfrequente persönliche Kontakt alleine scheint dabei jedoch nicht ausreichend zu sein, da selbst dreimal wöchentliche psychotherapeutisch basierte Therapiegespräche mit gleichzeitiger Naltrexonbehandlung zu schlechteren Ergebnissen führen als die Kombination mit Disulfiram [11].

In deutschsprachigen Anleitungen zum Vorgehen bei Beginn und Durchführung der Disulfiramtherapie wurde eine Standard Operation Procedure [19] mit zugehörigem Fortbildungsvideo [20] sowie das „Antabus-Coaching“ im Rahmen des Community Reinforcement Approach [12] publiziert.

Im Jahr 2011 stellte der einzige deutsche Hersteller den Vertrieb aufgrund nicht näher genannter „technischer Schwierigkeiten im Herstellungsprozess“ ein, sodass Disulfiram in Deutschland gegenwärtig keine Zulassung mehr hat. Dies wurde seitens der Deutschen Gesellschaft für Suchtforschung und Suchttherapie sowie der Deutschen Gesellschaft für Suchtmedizin als nicht hinnehmbare Behandlungslücke bedauert [8]. Eine Behandlung mit Disulfiram ist jedoch weiterhin legal möglich, indem es im Rahmen eines individuellen Heilversuches auf Privatrezept verschrieben und aus dem europäischen Ausland bezogen wird.

Prinzipiell sind hierzu alle Apotheken in der Lage, in der Praxis kommt es jedoch mitunter zu Lieferverzögerungen je nachdem, aus welchen Ländern die Pharmagroßhändler importieren und wie dort die aktuelle Verfügbarkeit ist. Beispiele für häufig lieferbare Präparate sind Esperal (Frankreich), Tetradin (Portugal), Refusal (Niederlande), Antabus (Schweiz, Spanien, Finnland) sowie Anticol (Polen). Bis vor einigen Jahren war als alternatives Medikament zur Inhibition der Acetaldehyd-Dehydrogenase Calciumcarbimid (Cyanamid) erhältlich (internationale Handelsnamen Kolme, Colme oder Temposil). Hierzu wurden in den letzten Jahrzehnten keine wissenschaftlichen Daten mehr publiziert.

Eine größere Anzahl von Suchtambulanzen psychiatrischer Kliniken hält dieses Therapieangebot auch seit 2011 weiter aufrecht bzw. führte es neu ein, da sie Disulfiram im Sinne der oben angeführten Leitlinienempfehlung als unverzichtbare Behandlungsoption für therapierefraktäre Patienten ansehen. Als „Off-Label“-Behandlung erfordert dies allerdings besondere Sorgfalt bei Indikationsstellung, rechtssicherer Patientenaufklärung sowie Dokumentation der Einwilligung. Um sich in diesen Fragen zu unterstützen, schlossen sich im Jahr 2018 dreizehn deutsche Einrichtungen im Rahmen eines Gründungsworkshops in Göttingen zum „Netzwerk alkoholaversive Pharmakotherapie“ (NAP) zusammen. Die naheliegenden Ziele des Netzwerks bestehen darin, eine hohe Behandlungsqualität zu gewährleisten und der Verunsicherung bezüglich rechtlicher Grundlagen entgegenzuwirken. Als Arbeitsmethode entwickelte sich ein elektronisches Kommunikationsforum, in dem patientenbezogene klinische Fragen einzelner Mitglieder gemeinsam diskutiert werden, wobei auch die einschlägige Literatur zusammengetragen wird. Einmal jährlich findet ein Workshop statt, bei dem häufig wiederkehrende Fragen vertieft diskutiert und die Ergebnisse als Expertenkonsens festgehalten werden. In diesem Zuge wurde auch eine rechtssichere Patientenaufklärung und Einwilligungserklärung erstellt. Aktuell gehören dem NAP 108 Fachärzte für Psychiatrie und Psychotherapie, Assistenzärzte, Psychologen, sozialpädagogische Suchttherapeuten sowie Gesundheits- und Krankenpfleger an, die an 48 Kliniken und 10 Arztpraxen in Deutschland sowie zwei schweizerischen, einer österreichischen und einer dänischen Einrichtung tätig sind. Durch die Beteiligung des Kopenhagener Zentrums konnte die Datenerhebung dieser Studie wesentlich erweitert werden, wenngleich die unterschiedlichen Rahmenbedingungen der Behandlung bei der Bewertung zu berücksichtigen sind. Dies betrifft vor allem die generell hohe Erwartungshaltung der dänischen Bevölkerung gegenüber Disulfiram als dem dort am häufigsten eingesetzten Medikament in der ambulanten Alkoholbehandlung.

Seit 2019 führt das Netzwerk bei seinen Mitgliedern jährliche Erhebungen zur Qualitätskontrolle durch, deren Methoden und Ergebnisse nachfolgend erstmals berichtet werden.

## Studiendesign und Untersuchungsmethoden

### Erhebungsinstrument

Die Erhebungen wurden in Form eines Fragebogens durchgeführt, um dessen Bearbeitung alle zum jeweiligen Zeitpunkt am Netzwerk teilnehmenden Einrichtungen gebeten wurden. Dabei wurde jeweils die Anzahl von Patienten abgefragt, auf die spezifische Aussagen zutrafen. Darüber hinaus bestanden keine Ein- oder Ausschlusskriterien. Zuletzt wurde dabei für den Bezugszeitraum jeweils des letzten Quartales nach der Patientenzahl, Trinkereignissen, psychiatrischer Komorbidität, Einnahme von Psychopharmaka und Teilnahme an Gruppentherapien gefragt. Für den Bezugszeitraum des jeweils letzten Jahres wurden Nebenwirkungen, stationäre Aufnahmen wegen Aversivreaktionen und schwere unerwünschte Ereignisse erfasst. Die Erhebungen wurden mit einer pandemiebedingten Ausnahme 2020 jährlich durchgeführt, wobei die Versionen von 2019 und 2021 nicht alle der oben genannten Fragen enthielten. Die hier ausgewerteten Fälle wurden von den im elektronischen Supplement genannten 33 Einrichtungen berichtet.

### Ethische Aspekte

Alle beschriebenen Untersuchungen wurden im Einklang mit nationalem Recht sowie gemäß der Deklaration von Helsinki von 1975 (in der aktuellen, überarbeiteten Fassung) durchgeführt. Die Behandlung der Patienten erfolgte ausschließlich gemäß individuellen klinischen Erwägungen und wurde nicht durch die hier durchgeführte Erhebung beeinflusst. Da also nicht studienbedingt in die psychische oder körperliche Integrität eines Menschen eingegriffen wurde und es aufgrund der aggregierten Datenübermittlung nicht möglich war, diese auf einzelne Menschen zuzuordnen, wurde in Übereinstimmung mit § 15 der Berufsordnung für Ärzte Bayerns keine Beratung durch eine Ethikkommission durchgeführt.

## Ergebnisse

### Patientenmerkmale

In der Summe der Jahre 2019 bis 2023 wurden insgesamt 1579 Behandlungsfälle beschrieben. Mit hoher Wahrscheinlichkeit wurde ein Teil der Patienten im Längsschnitt über mehr als ein Jahr behandelt und ging somit mehrfach in die Auswertung mit ein. Wie hoch dieser Anteil ist lässt sich nicht zuverlässig abschätzen, da die Daten nicht auf einzelne Patienten, sondern auf das jeweilige Zentrum bezogen erhoben wurden. Die Fälle ließen sich wie folgt näher charakterisieren: 1108 (70 %) Männer, 471 (30 %) Frauen, 106 (7 %) in gleichzeitiger Opioidsubstitution, 18 (1 %) in forensischem Behandlungskontext, 237 (15 %) Eltern minderjähriger Kinder, 132 (8 %) über 64-Jährige und 14 (1 %) intelligenzgeminderte Patienten.

### Trinkereignisse und Aversivreaktionen

**Abb. 1:**
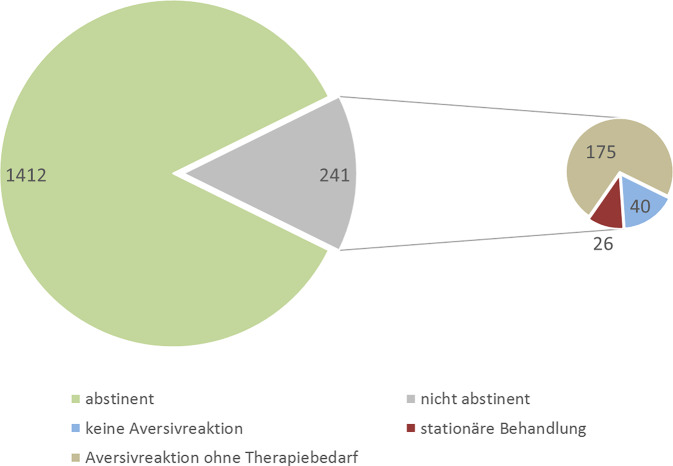
Kumulative Inzidenz und Verlauf von Alkoholaversivreaktionen 2019 bis 2023

Wie aus Abb. 1 ersichtlich ist, wurden bei 152 Patienten insgesamt 241 Trinkereignisse erfasst. In der überwiegenden Mehrheit von 73 % aller Fälle war dies für die Patienten kein Anlass, medizinische Hilfe in Anspruch zu nehmen. Bei 11 % der Trinkereignisse wurden die Patienten für mehrere Stunden stationär überwacht, wobei kein Fall von intensivmedizinischem Überwachungs- oder Therapiebedarf berichtet wurde. In 16 % der Fälle kam es zu keiner Aversivreaktion.

### Nebenwirkungen von Disulfiram

Bezüglich der am häufigsten zu erwartenden Nebenwirkungen wurde in den Fragebögen explizit erfragt, welche Symptome während der Behandlung mit Disulfiram erstmals auftraten bzw. sich deutlich verschlechterten, ob dies zum Absetzen von Disulfiram führte und ggf. ob sich die Nebenwirkungen nach Absetzen zurückbildeten. Die Ergebnisse sind in Tab. 1 dargestellt. Der dort als persistierend beschriebene Fall einer Polyneuropathie hatte bereits vor Behandlungsbeginn mit Disulfiram bestanden und sich weiter verschlechtert.

**Tab. 1:** Kumulative Inzidenz und Verlauf häufiger Nebenwirkungen von Disulfiram 2019 bis 2023

	Häufigkeit (% aller Patienten)	Deshalb abgesetzt	Nicht reversibel
Mund‑/Körpergeruch	31 (2,5)	4	1
Müdigkeit	28 (2,3)	2	0
Männliche sexuelle Funktionsstörungen	17 (1,4)	4	0
Transaminasenanstieg	15 (1,2)	6	0
Allergische Hautreaktionen	13 (1,1)	5	1
Polyneuropathie	10 (0,8)	4	1
Klinisch relevante Hepatitis	1 (0,08)	1	1

An schweren unerwünschten Ereignissen ist ein Fall einer perakut beginnenden Hepatitis bei einer nicht opiatsubstituierten Patientin in der 10. Behandlungswoche mit Disulfiram 125 mg täglich hervorzuheben, in deren Verlauf trotz des Absetzens von Disulfiram eine Lebertransplantation erforderlich wurde. Ein Kausalzusammenhang mit Disulfiram ließ sich zwar nicht nachweisen, ist jedoch wahrscheinlich. In einem weiteren Fall trat während der Behandlung mit Disulfiram erstmals eine axonale sensomotorische Polyneuropathie der Beine auf, die nach Absetzen nur leicht rückläufig war. Nach ausführlicher elektrophysiologischer Diagnostik wurde seitens der behandelnden Neurologen ein funktioneller Anteil angenommen und ein Kausalzusammenhang mit Disulfiram als unwahrscheinlich eingeschätzt. An weiteren, nicht systematisch erfragten Nebenwirkungen wurden Übelkeit (0,8 %), Kopfschmerzen (0,4 %) sowie vereinzelt Diarrhö, isolierter Appetitmangel und Lipaseerhöhung ohne klinisches Korrelat berichtet. Die gezielte Frage nach weiblichen sexuellen Funktionsstörungen wurde in keinem Fall bejaht. Bei den beiden berichteten Todesfällen war kein Kausalzusammenhang zur Disulfirambehandlung erkennbar, insbesondere standen sie sicher nicht mit einer Aversivreaktion im Zusammenhang.

### Prävalenz komorbider Sucht- und anderer psychiatrischer Erkrankungen

**Abb. 2:**
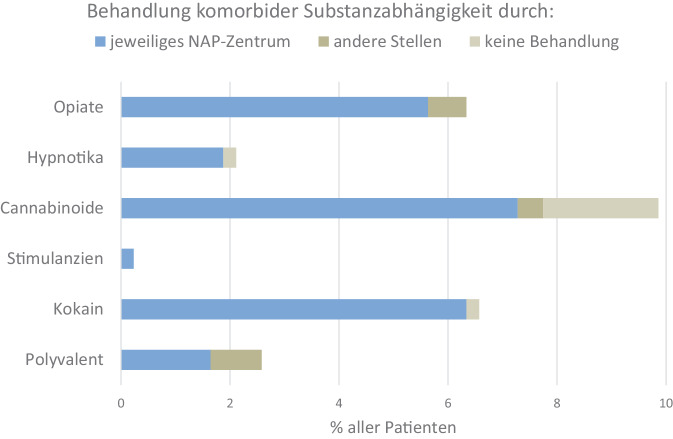
Prävalenz weiterer Abhängigkeitserkrankungen gemäß ICD-10 und Anteil deren Behandlung bei verschiedenen Stellen im Jahr 2023. *NAP* Netzwerk alkoholaversive Pharmakotherapie

Angaben zu komorbiden psychiatrischen Erkrankungen wurden erst ab 2022 systematisch erhoben und sind hier für die 426 Patienten dargestellt, die im Jahr 2023 berichtet wurden (Abb. 2). Bei einem nennenswerten Anteil der Patienten lag gleichzeitig eine weitere Suchterkrankung neben der Alkoholabhängigkeit vor, die zumeist von derselben Einrichtung behandelt wurde, die auch das Disulfiram vergab. Die opiatabhängigen Patienten wurden dabei mit wenigen Ausnahmen substituiert, während die Behandlung bei Abhängigkeit von Hypnotika, Stimulanzien, Kokain oder polyvalentem Konsummuster aufgrund der potenziell gefährlichen Interaktion mit einer Alkoholaversivreaktion strikt abstinenzorientiert erfolgte. Auch bei den cannabisabhängigen Patienten wurde auf Abstinenzmotivation hingearbeitet, tatsächliche Abstinenz war jedoch nicht in allen Zentren Voraussetzung für Beginn bzw. Aufrechterhaltung der Therapie mit Disulfiram.

**Abb. 3:**
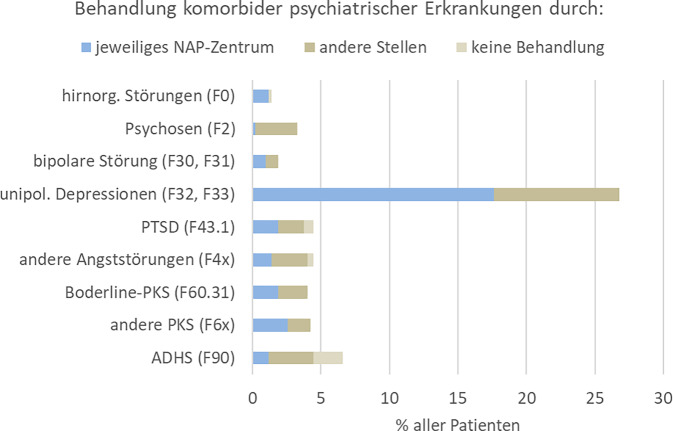
Prävalenz komorbider psychiatrischer Erkrankungen und Anteil deren Behandlung bei verschiedenen Stellen im Jahr 2023. *NAP* Netzwerk alkoholaversive Pharmakotherapie, *PTSD* posttraumatische Belastungsstörung, *PKS* Persönlichkeitsstörung, *ADHS* Aufmerksamkeitsdefizit‑/Hyperaktivitätsstörung

Neben stoffgebundener Abhängigkeit traten erwartungsgemäß auch andere psychiatrische Störungen komorbid auf, vor allem unipolare Depressionen. Ihre Häufigkeit und Behandlungssettings sind in Abb. 3 dargestellt.

### Einbettung der Behandlung mit Disulfiram in ein Gesamttherapiekonzept

Die Behandlung der Patienten bestand überwiegend nicht nur in der Gabe von Disulfiram, sondern schloss eine multimodale Therapie der Alkoholabhängigkeit und komorbider psychiatrischer inklusive suchtbezogener Erkrankungen mit ein. Unter anderem wurde eine psychopharmakologische Behandlung durchgeführt, welche bei Auswertung der 426 Patienten des Jahres 2023 in absteigender Häufigkeit die Gabe folgender Medikamente umfasste: Antidepressiva (32 % aller Patienten), sedierende Antipsychotika ohne Vorliegen einer Psychose (12 %), Opiatsubstitutionsmittel (7 %), Naltrexon (5 %), Antipsychotika zur Psychosetherapie (5 %), Benzodiazepine (4 %), Stimulanzien bei ADHS (3 %) und Stimmungsstabilisatoren (2 %).

**Abb. 4:**
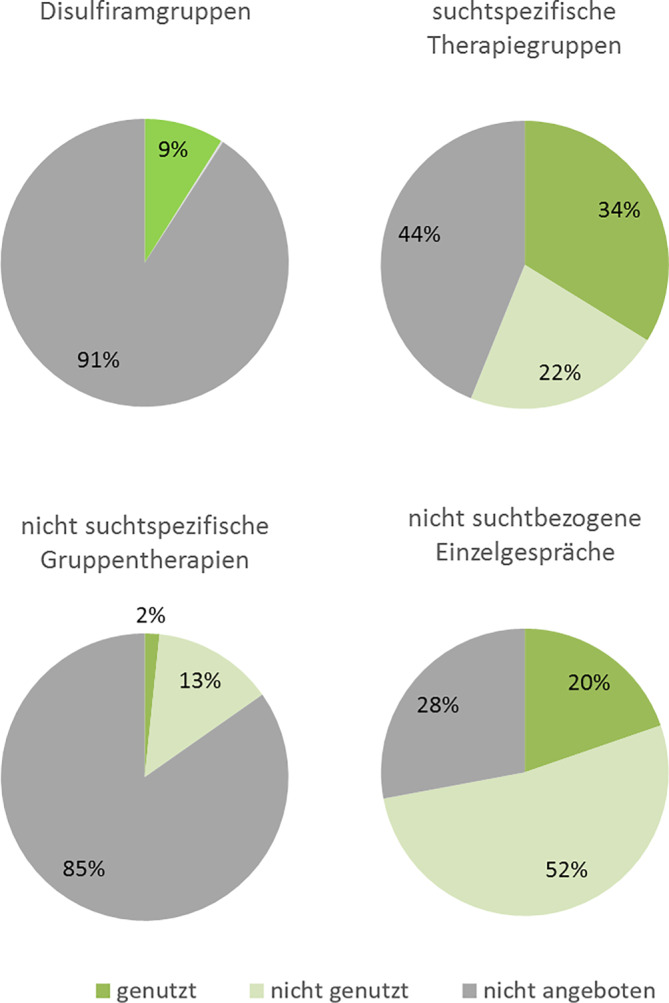
Prozentualer Anteil der Patienten, welche im Jahr 2023 weitere Therapieformen der NAP (Netzwerk alkoholaversive Pharmakotherapie)-Zentren wahrnahmen, nicht wahrnahmen oder nicht angeboten bekamen

Daneben wurden seitens der NAP-Zentren auch – abhängig von deren personellen und räumlichen Ressourcen – weitere Therapieformen angeboten (Abb. 4). Dazu gehörten das Antabus-Coaching und Abstinenzkonto im Rahmen des Community Reinforcement Approach [18], Disulfiramgruppen, an denen Patienten ausschließlich während einer Behandlung mit diesem Medikament teilnehmen konnten, sowie weitere suchtspezifische Therapiegruppen, die auch Patienten ohne Disulfirambehandlung offenstanden. Anbieter dieser Gruppen waren Psychologen, Sozialpädagogen, Ärzte und Pflegepersonal. Zusätzlich wurden mancherorts seitens Bewegungs‑, Ergo- oder Ernährungstherapeuten allgemeine Gruppentherapien angeboten, deren Fokus nicht spezifisch auf Suchtthemen lag. Insgesamt erhielten 64 % aller Patienten das Angebot mindestens eines dieser Therapieangebote, das zu den in Abb. 4 dargestellten Anteilen wahrgenommen wurde. Des Weiteren bestand für nahezu drei Viertel der Patienten zusätzlich das Angebot psychotherapeutisch basierter ärztlicher, psychologischer, sozialpädagogischer oder pflegerischer Einzelgespräche zur Therapie nicht suchtbezogener psychiatrischer Komorbidität.

## Diskussion

Mit der Beschreibung von über 1500 Behandlungsfällen gelang es dem NAP, den unserer Kenntnis nach bisher umfangreichsten Einzelbericht zu Patientencharakteristika, Nebenwirkungen, Behandlungssetting sowie zu Häufigkeit und Folgen von Alkoholaversivreaktionen zusammenzutragen. Gerade bezüglich Aversivreaktionen kann die Erkenntnis über deren insgesamt mäßige Konsequenzen dazu beitragen, die bei Therapeuten und Patienten häufig vorherrschenden Vorstellungen von lebensgefährlichen oder gar tödlichen Folgen zu relativieren und diesem Therapiehindernis entgegenzuwirken. Auch die Häufigkeit von Trinkereignissen während der Disulfirambehandlung hält sich in überschaubaren Grenzen. Warum es bei einem Sechstel der Trinkereignisse zu keiner nennenswerten Aversivreaktion kam, könnte einerseits dadurch erklärt werden, dass diese Patienten das Disulfiram einige Tage zuvor absichtsvoll abgesetzt hatten, um trinken zu können. Andererseits kann es vorkommen, dass die Prodrug Disulfiram, abhängig von genetischen Polymorphismen der beteiligten Enzyme oder anderen pharmakologischen Ursachen, nicht ausreichend in die aktive Form von Diethylthiomethylcarbaminsäuremethylester (DDTC-Me) und andere aktive Metabolite umgewandelt wird [3]. Bei den davon betroffenen Personen kann eine Aversivreaktion somit nur durch ungewöhnlich hohe Disulfiramdosierungen bzw. gar nicht ausgelöst werden. Dies könnte anhand von Plasmaspiegelbestimmungen von DDTC-Me erkannt und die Dosierung daran ausgerichtet werden [3].

Bezüglich der hier beobachteten Nebenwirkungen fällt auf, dass diese teils von den in der letzten verfügbaren Fachinformation zu Antabus-Dispergetten vom Juli 2007 beschriebenen [13] abweichen. Die dort als „sehr häufig“ angegebenen Nebenwirkungen Müdigkeit, Mund‑/Körpergeruch, Oberbauchbeschwerden und Schweregefühl im Kopf traten bei unserer Erhebung deutlich seltener auf. Dies könnte mit der überwiegend langfristigen Disulfirameinnahme zusammenhängen: Entweder stellt diese Gruppe eine Positivauslese von Patienten dar, welche die Behandlung nicht wegen dieser Nebenwirkungen abbrach, oder es handelt sich hier um frühe Nebenwirkungen, die bei langdauernder Einnahme nachlassen. Alternativ könnten seltener auftretende Nebenwirkungen auch auf die heutzutage eher niedrigeren Dosierungen zurückzuführen sein. Die besser objektivierbaren Nebenwirkungen von Polyneuropathie, allergischen Hautreaktionen und benignem Transaminasenanstieg traten bei den hier beschriebenen Patienten gleich häufig oder minimal öfter auf als in der Fachinformation beschrieben. Dies spricht dafür, dass unsere Erhebungsmethode die auftretenden Nebenwirkungen tatsächlich sensitiv erfasste und nicht systematisch unterschätzte. Dem genannten Risiko peripherer Polyneuropathien steht laut einer translationalen Studie womöglich ein protektiver Einfluss von Disulfiram auf die Entwicklung der Demenz vom Alzheimer-Typ entgegen [14].

Die unsererseits mit 1,4 % als „häufig“ einzuschätzenden männlichen sexuellen Funktionsstörungen wurden in der Fachinformation nicht als Nebenwirkung aufgeführt. Dagegen traten die dort „gelegentlich“, also immerhin bei jedem hundertsten bis tausendsten Patienten zu erwartenden Depressionen, Verwirrtheitszustände, maniformen und paranoid-halluzinatorischen Psychosen in unserem Patientenkollektiv nicht auf. Der tragische Fall einer akut aufgetretenen Hepatitis mit Lebertransplantation wäre laut zwei großen Registerstudien statistisch erst nach mindestens 30.000 behandelten Patienten zu erwarten gewesen [2, 6].

Bei zwei Dritteln der Patienten beinhaltete die Behandlung neben Disulfiram auch Gruppentherapieangebote, welche von diesen Patienten zu 70 % auch wahrgenommen wurden. Therapiegespräche im Einzelsetting wurden 72 % aller Patienten angeboten, von diesen jedoch nur zu 27 % genutzt. Die Psychopharmakotherapie als weitere Behandlungsmodalität zeigte sich in Kombination mit Disulfiram gut verträglich, Interaktionen oder Komplikationen im Falle von Aversivreaktionen wurden nicht beobachtet. Die durch Disulfiram „erzwungene“ Abstinenz stellte somit für die Mehrheit der Patienten lediglich sicher, dass die multimodalen Therapieangebote inklusive der beschriebenen Psychopharmakotherapie konsequent in Anspruch genommen werden und somit ihre Wirksamkeit entfalten konnten. Ein solches Gesamttherapiekonzept stellt eine wesentliche Weiterentwicklung gegenüber der früher üblichen isolierten Rezeptierung von Disulfiram als einziger Behandlungsmaßnahme dar.

Aufgrund fehlender Förderung konnte diese Erhebung nur in Form einer naturalistischen Begleituntersuchung ohne prospektive Datenerhebung durchgeführt werden. Auch eine Analyse auf Einzelfallebene war deshalb nicht möglich, sodass leider unklar bleiben muss, auf wie vielen Patienten die 1579 Behandlungsfälle eigentlich basieren. Diesen methodischen Schwächen steht als Stärke die große Zahl berichteter Fälle gegenüber sowie die Tatsache, dass die große Mehrheit davon unter den realen Alltagsbedingungen von Versorgungskliniken behandelt wurde. Diese Ergebnisse können somit als repräsentativ angesehen werden und lassen sich ohne Weiteres auf andere Zentren extrapolieren.

## Fazit für die Praxis

Trotz fehlender deutscher Zulassung bewährt sich aus dem europäischen Ausland bezogenes Disulfiram weiterhin als wertvolle Therapieoption, besonders für therapieresistente und hoch rückfallgefährdete Patienten. Aversivreaktionen führen in der Regel nur zu mäßigen Symptomen, lebensbedrohliche Verläufe wurden hier nicht beobachtet. Im Rahmen eines individuellen Heilversuches lässt sich die Behandlung rechtssicher, nebenwirkungs- und komplikationsarm durchführen. Sie kann von Ambulanzen psychiatrischer Kliniken sowie Praxen gut als Bestandteil eines multimodalen Therapiekonzeptes angeboten werden. Das deutschsprachige Netzwerk alkoholaversive Pharmakotherapie hat sich in den letzten Jahren als wertvolle Hilfe zur Entscheidungsfindung bei schwierigen klinischen Einzelfragen bewährt und unterstützte mehrere psychiatrische Zentren sowie Fachpraxen dabei, die Behandlung mit Disulfiram neu zu etablieren. Eine Wiederzulassung von Disulfiram in Deutschland erscheint dringlich geboten.

## Supplementary Information

Beschreibung der teilnehmenden Zentren
