# Supplementary material for: Patient and quality characteristics in the treatment with disulfiram (Antabus) in the German “Network for Alcohol Aversive Pharmacotherapy”
Source: Nervenarzt. 2024 Aug 22;96(2):159–65. [Article in German] doi: 10.1007/s00115-024-01714-5 (PMC11876189; doi:10.1007/s00115-024-01714-5)
Supplement: Supplementary file 1 — Beschreibung der teilnehmenden Zentren [file 115_2024_1714_MOESM1_ESM.pdf]

Patienten- und Qualitätsmerkmale bei der Behandlung mit Disulfiram („Antabus“) im deutschsprachigen Netzwerk alkoholaversive Pharmakotherapie

Ulrich S. Zimmermann 1,2, Clemens Plickert 3, Christel Lüdecke 4, Markus Stuppe 5, Christian Rosenbeiger 1, Yvonne Krisam 6, Tobias Link 7, Jean Keller 7, Gero Bühler 8, Deborah Scholz-Hehn 9, Ursula Havemann-Reinecke 10, Dirk Wedekind 10, Mathias Luderer 11, Maik Spreer 2, 12

Elektronisches Supplement

Beschreibung der teilnehmenden Zentren

In Summe der vier Erhebungszeitpunkte trugen folgende Zentren die jeweils in Klammern genannte Anzahl von Fällen bei: Center for Alkohol og Stofbehandling Kopenhagen (391), Bezirkskrankenhaus Kempten (186), Asklepios Klinikum Göttingen (168), Universitätsklinikum Dresden (83), Helios Klinikum Schwerin (81), LVR-Klinik Langenfeld (80), kbo Isar-Amper-Klinikum, Klinik für Suchtmedizin und Psychotherapie in Haar und Evangelisches Klinikum Bielefeld-Bethel (je 66), kbo Isar-Amper-Klinikum München Nord (60), Zentralinstitut für Seelische Gesundheit Mannheim (57), LWL-Klinik Münster (48), Psychiatrisches Zentrum Nordbaden Wiesloch (46), Klinika Ingolstadt und Karlsruhe (je 38), Klinikum Oberberg Bergisch Gladbach (23), St. Valentinus-Krankenhaus Kiedrich (20), LWL-Klinikum Gütersloh (17), kbo Isar-Amper-Klinikum, Klinik für forensische Psychiatrie (15), Universitätsklinikum Hamburg (13), Ameos Klinikum Osnabrück (11), Diakonie-Krankenhaus Elbingerode (11), Zentrum für Psychiatrie Ravensburg (10), Bezirkskrankenhaus Regensburg (9), Universitätsmedizin Göttingen (9), LMU-Klinikum München (9), LWL-Klinikum Dortmund (7), Suchtpraxis Lüneburg (6), Universitätsklinikum Frankfurt/M (4), LVR-Klinik Essen (3) sowie Praxis für Allgemeinmedizin Berlin, Agaplesion Markus-Krankenhaus Frankfurt/M, Elblandklinik Radebeul, AMEOS Klinik für forensische Psychiatrie Neustadt/Holstein (je 1). 13 dieser Zentren trugen Daten zu allen vier Erhebungszeitpunkten bei, 6 zu drei Zeitpunkten, 6 zu zwei Zeitpunkten und 8 nur zu einem.

Die Anzahl der pro Quartal in den einzelnen Zentren gleichzeitig behandelten Patienten betrug im Median 12, wobei die drei größten deutschen Zentren jeweils maximal 51, 46 und 32 und die drei kleinsten Zentren jeweils nur einen Patienten gleichzeitig behandelten. In Kopenhagen als dem insgesamt größten Zentrum wurden pro Quartal zwischen 179 und 212 Patienten gleichzeitig behandelt.
